# Cardiac structure and function across the continuum of glucose metabolism

**DOI:** 10.14814/phy2.70429

**Published:** 2025-06-17

**Authors:** Tommi Grönlund, Kari Kaikkonen, M. Juhani Junttila, Olavi Ukkola, Risto Kerkelä, Heikki V. Huikuri, Mikko P. Tulppo

**Affiliations:** ^1^ Research Unit of Biomedicine and Internal Medicine University of Oulu Oulu Finland; ^2^ Medical Research Center Oulu, Oulu University Hospital and University of Oulu Oulu Finland

**Keywords:** cohort analysis, echocardiography, HbA1c, prediabetes, systolic function

## Abstract

Diabetes and prediabetes increase the risk of heart failure, but the relationship between glycated hemoglobin (HbA1c) and left ventricular systolic function in the general population is not known. This study aimed to investigate the relationship between HbA1c and global longitudinal strain (GLS) in nondiabetic and prediabetic subjects. A subpopulation of the Northern Finland Birth Cohort 1966 took part in a follow‐up, including extensive medical examination and echocardiography (*n* = 1155), at the age of 46. The final study population included 636 healthy subjects. Normal HbA1c levels were divided into sex‐specific tertiles and prediabetes, which were later pooled together. Univariate analysis after adjusted with covariates was used to compare cardiac function in HbA1c groups. HbA1c groups were defined as low (30.9 ± 1.7 mmol/mol, *n* = 190), medium (34.6 ± 1.0 mmol/mol, *n* = 189), high (37.4 ± 1.0 mmol/mol, *n* = 190) and prediabetes (41.0 ± 1.3 mmol/mol, *n* = 67) groups. Subjects with abnormal absolute GLS (<18) comprised 7.4%, 12.7%, 21.1%, and 17.9% (main effect *p* < 0.01) of low, medium, high HbA1c and prediabetes groups, respectively. HbA1c was not associated with any cardiac structure variables. High normal HbA1c levels and prediabetes are associated with reduced myocardial contractility in a healthy middle‐aged population, potentially predicting the development of heart failure.

## Graphical abstract

Myocardial contractility and blood glucose

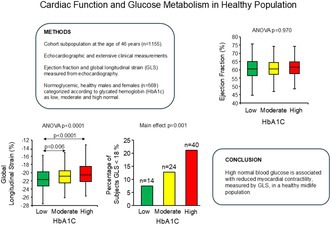

## 1 INTRODUCTION

Cardiovascular diseases (CVDs) are the leading cause of death globally, accounting for 32% of all global deaths (World Health Organization, 2023a). Type 2 diabetes (T2DM) is a major risk factor for CVD, and its prevalence has been predicted to rise from 529 million patients in 2021 to 1.31 billion by 2050, increasing the burden on healthcare systems significantly (World Health Organization, 2023b). This trend highlights the importance of identifying and managing prediabetes, an intermediary metabolic state associated with elevated risk for cardiovascular events and all‐cause mortality (Mando et al., 2021). Because CVDs and type 2 diabetes share common modifiable behavioral risk factors, such as diet, exercise, smoking, and alcohol use (Lawal et al., 2020), even earlier interventions and preventive measures are crucial for reducing the overall disease burden of these interlinked diseases (Ong et al., 2023).

Glycated hemoglobin (Hba1c) is an important biomarker that indicates plasma glucose levels over the prior 2–3 months (Sargowo, 2020). The American Diabetes Association (ADA) has recommended Hba1c as the most important prognostic and diagnostic tool for diabetes (Elsayed et al., 2023). Elevated Hba1c levels are linked to CVDs such as ischemic heart disease and heart failure, even in individuals without diabetes (Cavero‐Redondo et al., 2017). Chronic hyperglycemia can lead to an array of macrovascular and microvascular complications and, further, to diabetic cardiomyopathy (Dillmann, 2019; Škrha et al., 2016).

Cardiac function is commonly evaluated in clinical practice using left ventricular ejection fraction (LVEF). However, in recent years echocardiographic deformation measures such as global longitudinal strain (GLS) have been suggested to be more sensitive than LVEF at detecting LV dysfunction (Potter & Marwick, 2018). GLS is calculated as the combined deformity (strain) of 18 segments of the left ventricle and expressed as a percentage. It represents the lengthening and shortening of myocardial fibers in the longitudinal axis. GLS reflects predominantly the function of the subendocardial and longitudinal myocardial fibers, which are the most susceptible to ischemic damage and wall stress (Campbell et al., 2019). Reduced absolute GLS can be a sign of subclinical ventricular dysfunction even while LV ejection fraction remains within normal ranges. Recently, GLS has gained recognition as a well‐validated and reproducible tool for evaluating cardiac contractility and has been shown to provide additional prognostic value over LVEF at predicting several cardiac events and complications (Sengeløv et al., 2015). The relationship in the middle‐aged general population between glycemic control and modern cardiac function measures such as GLS and the ratio of early diastolic mitral inflow velocity (E) to early diastolic mitral annulus velocity (e') (known as E/e') is not well known.

## 2 RESEARCH DESIGN AND METHODS

### 2.1 Study participants

The subjects are a subpopulation of the Northern Finland Birth Cohort 1966 (NFBC1966) (University of Oulu, 1966). The NFBC1966, a large prospective general population‐based longitudinal birth cohort, includes 96.3% of all live births (*n* = 12,058) in 1966 in the two northernmost provinces of Finland. Detailed information about the NFBC1966 has been previously described elsewhere (Nordström et al., 2021). The study was conducted according to the Declaration of Helsinki and approved by the ethical committee of the Northern Ostrobothnia Hospital District in Oulu, Finland. Written informed consent was received from all the participants.

### 2.2 Protocol

Health status and lifestyle were determined by sending postal questionnaires to the participants with known addresses in Finland. An invitation to clinical examinations was sent to a total of *n* = 10,282 subjects with known addresses in Finland at age 46. Between April 2012 and March 2014, 5861 subjects (57%) participated in clinical examinations in one of three laboratory units (Oulu, Southern, and Northern Finland). Before the examinations, the participants were told to abstain from smoking and drinking caffeinated beverages. The clinical examinations consisted of anthropometric measures, blood sampling, cardiovascular health status assessment, and a 2‐h glucose tolerance test conducted on a separate day after a 12‐h fast. Glucose and lipid profiles were analyzed from venous blood samples. The diagnosis of diabetes was made in line with the criteria of the World Health Organization: fasting plasma glucose ≥7.0 mmol/L, or 2‐hour glucose in oral glucose test ≥11.1 mmol/L, or glycated hemoglobin ≥6.5% (Alberti & Zimmet, 1997). Systolic and diastolic blood pressures were measured three times with participants in a seated position after 15 min of rest. (2 of the 3 lowest values were averaged). The device used was an automated sphygmomanometer (Omron M10; Omron Healthcare, Kyoto, Japan).

### 2.3 Echocardiographic measurements

Echocardiographic examinations were conducted for a randomly selected subpopulation, *n* = 1155 (19.7%). An experienced cardiologist (K.K.) performed a comprehensive transthoracic 2‐dimensional echocardiography using a General Electric Vivid E9 device with an M5S‐D 1.5/4.6 MHz sector transducer for cardiovascular imaging (GE Health Medical, Horten, Norway). The measurements were performed according to the guidelines of the American Society of Echocardiography (Lang et al., 2006). In our assessment of cardiac structure and function, we focused on the following echocardiographic variables: LV mass, LV end‐diastolic volume, interventricular septal thickness, posterior wall thickness, relative wall thickness (RWT), left atrial (LA) end‐systolic volume, left ventricle ejection fraction (LVEF), and the ratio of early diastolic mitral inflow velocity to early diastolic mitral annulus velocity as a marker of diastolic function (E/e'). Global longitudinal strain as a marker of myocardial deformity was later calculated using Echopac 7 software (automated function imaging). Based on ASE/EACVI guidelines and vendor‐specific data for GE devices, a GLS cutoff value of <18% was used to define abnormal LV longitudinal strain (Lang et al., 2015). RWT was calculated as two times posterior wall thickness divided by LV diastolic diameter (Foppa et al., 2005). Measures of the cardiac structure were indexed to body surface area (the Dubois equation).

### 2.4 Clinical and anthropometric measures

All anthropometric measurements were performed after an overnight fast of 12 h. Body weight was measured with a regularly calibrated digital scale. Height was measured using a standard and calibrated stadiometer. An average of two measurements was used. Body‐mass index (BMI) was calculated using the standard formula of weight (kg) divided by height (m) squared. Waist circumference was taken at the midlevel of the lowest rib margin and the iliac crest. Hip circumference was measured at the widest point of the trochanters. Both measurements were taken twice, and the average of the two was used. The waist‐to‐hip ratio was later derived from these circumferences. An automated blood pressure device (Omron Digital Automatic Blood Pressure Monitor Model M10‐IT) was used for blood pressure measurements. Brachial systolic (SBP) and diastolic (DBP) blood pressures were measured in a seated position from the right arm. Each measurement was taken three times at 1‐min intervals after 15 min of rest. Finally, the average of the two lowest systolic values and corresponding diastolic values were used.

### 2.5 Laboratory measurements

The blood samples were taken after an overnight fast and then immediately centrifuged and analyzed without storing. The blood samples were analyzed in the NordLab Oulu testing laboratory (T113) accredited by the Finnish Accreditation Service (FINAS) (EN ISO 15189). Hba1c was analyzed between 7.00 and 11.00 after an overnight fast using immunochemical assay methods (ref 10485591) (all methods by Advia 1800; Siemens Healthcare Diagnostics Inc., Tarrytown, NY, USA). Fasting serum insulin (fs‐Insulin, ref. 02230141) and fasting blood glucose (fB‐glucose, ref. 05001429) levels were assessed by radioimmunoassay (Pharmacia Diagnostics, Uppsala, Sweden) and further analyzed using an enzymatic dehydrogenase method (Advia 1800, Siemens Healthcare Diagnostics, Tarrytown, NY, USA) and by a chemiluminometric immunoassay (Advia Centaur XP, Siemens Healthcare Diagnostics, Tarrytown, NY, USA). Homeostatic model assessment of insulin resistance (HOMA‐IR) was used to evaluate insulin resistance. HOMA‐IR was calculated as (fB‐glucose × fs‐insulin/22.5). Total cholesterol (ref 10376501), high‐density lipoprotein (HDL, ref. 07511947) and low‐density lipoprotein cholesterol (LDL, ref. 09796248), and triglycerides (ref 09580156) were determined using an enzymatic assay method.

### 2.6 Lifestyle factors

Participants' smoking and drinking habits were assessed based on answers to the questionnaire at the age of 46. “Current smoker” was defined as ≥1 cigarette per day on $\geq 2\;d∙{\mathrm{wk}}^{-1}$. Daily alcohol consumption was estimated based on the self‐reported frequency and number of alcoholic beverages consumed. The subjects were categorized into three alcohol consumption groups according to Finnish Institute for Health and Welfare (THL) alcohol consumption risk levels. The cutoff values were as follows: 10 g∙*d* ^−1^ (moderate) and 20 g∙*d* ^−1^ (high) for women, and 20 g∙*d* ^−1^ (moderate) and 40 g∙*d* ^−1^ (high) for men. Total sitting time was estimated based on the self‐reported sitting hours during weekdays (at work, home, commuting/in a vehicle, or elsewhere). The total sum of sitting time was then dichotomized using a previously established cutoff value of 11 $h∙d^{-1}$(van der Ploeg et al., 2012). Insufficient sleep was determined by asking the subjects if they typically felt tired in the morning within 30 min of waking up. The answer “very/somewhat tired” was defined as insufficient, and “somewhat/well‐rested” was defined as sufficient sleep.

### 2.7 Inclusion/exclusion

Among the subjects with echocardiographic data (*n* = 1155), participants with a missing value of GLS (*n* = 194), suboptimal quality of echocardiography (*n* = 87), heart rate (during echocardiography) over 85 bpm (*n* = 105), any significant echocardiographic abnormality (*n* = 21), cardiac disease (*n* = 24), respiratory disease (*n* = 61), and diabetes (*n* = 27) were excluded. The final study population included 636 subjects, 289 (45.4%) men and 347 (54.6%) women.

### 2.8 Statistical analysis

The data were analyzed using SPSS software (IBM SPSS Statistics 28; IBM Corp, Armonk, New York). A *p* value of <0.05 was considered significant, and later, Bonferroni correction was used to account for multiple testing. The dependent variables were checked for normality (Gaussian distribution) by visual inspection. One‐way analysis of variance (ANOVA) was used to compare Hba1c tertiles, prediabetes, and the sexes. Bonferroni's post hoc test was used when applicable. No significant interaction between the sexes was found. Thus, men and women were analyzed conjointly. Multivariate linear regression (enter method) was used to estimate the relationship between dependent variables, independent variables, and possible confounding variables. Based on literature the following variables were considered for the enter method: Sex, BMI, Hba1c, SBP, HDL, total cholesterol, alcohol consumption, and smoking. Additionally, the most statistically significant predictors of GLS and E/e' were identified using stepwise method multivariate linear regression. Further, a hierarchical model system was used to compare the effect of the most important predictor variables and the two models (enter and stepwise). The relationships between the predictor variables and the outcome variables were checked for linearity by visually inspecting the scatterplots. The assumption of homoscedasticity was checked by generating scatterplots of standardized residuals against standardized predictor values. The scatterplots showed a random scatter of points, indicating homoscedasticity. The residuals were checked for normality using visual inspection of histograms of residuals and Normal P–P Plots of standardized regression residuals. To control for possible multicollinearity, predictor variables with tolerance lower than 0.2 or variance inflation factor over 5 were excluded.

## 3 RESULTS

### 3.1 Demographics and clinical characteristics

The demographics and clinical characteristics of subjects across the three Hba1c tertiles (low, medium, and high) are shown in Table 1. Subjects with higher HbA1c levels tended to have higher BMI and waist‐to‐hip ratio.

**TABLE 1:** Characteristics of study population within Hba1c tertiles. One‐way ANOVA or Chi‐Square tests were used to compare the groups.

	HbA1c tertiles			PreDM	*p*‐level
	Low	Medium	High		
	*n* = 190	*n* = 189	*n* = 189	*n* = 67	
Antropometrics					
Male, *n* (%)	85 (45)	85 (45)	85 (45)	34 (51)	0.837
Height, cm	172 (9)	171 (9)	172 (9)	172 (8)	0.733
Weight, kg	74 (14)	76 (14)	78 (14)	80 (15)	0.025
BMI, kg/m^2^	25.2 (3.4)	25.9 (3.8)	26.2 (3.9)	27.0 (4.5)**	0.003
Waist‐to‐hip ratio	0.88 (0.08)	0.89 (0.07)	0.90 (0.08)**	0.92 (0.08) **†	<0.001
Body fat, %	26.5 (7.6)	27.8 (8.5)	27.6 (8.4)	27.8 (8.2)	0.390
Metabolic syndrome, *n* (%)	20 (10.7)||	36 (19.7)	45 (24.3)	20 (30.3)||	<0.001
Medication					
Antihypertensive agents, *n* (%)	18 (9.5)	26 (13.8)	21 (11.1)	10 (14.9)	0.491
Anticholesterol agents, *n* (%)	1 (0.5)	9 (4.8)	6 (3.2)	2 (3.0)	0.097
Lifestyle					
Current smoker, %	15 (8.0)	25 (13.7)	26 (14.4)	12 (18.2)	0.106
Alcohol consumption, g/d	9.8 (17.0)	10.1 (15.5)	11.3 (17.5)	8.9 (13.8)	0.718
Alcohol risk use, *n* (%)	12 (6.4)	18 (9.9)	18 (9.8)	4 (6.1)	0.492
Glucose metabolism					
Hba1c, mmol/mol	30.9 (1.7)	34.6 (1.0)**	37.4 (1.0)** †	41.0 (1.3) **†	<0.001
Hba1c %	5.0 (0.2)	5.3 (0.1)	5.6 (0.1)	5.9 (0.1)	<0.001
F‐glucose, mmol/L	5.1 (0.4)	5.3 (0.5)	5.4 (0.5)**	5.7 (0.5) **†	<0.001
F‐insulin, mU/L	6.0 (3.0)	7.0 (3.9)	7.8 (5.4)**	8.7 (5.2) **†	<0.001
HOMA‐IR^a^	1.3 (1.1)	1.7 (1.4)	1.7 (1.6)	2.1 (1.9)	<0.001
HOMA‐B^a^	69 (44)	76 (49)	72 (49)	80 (57)	0.135
Laboratory measurements					
SBP, mmHg	122 (15)	123 (16)	124 (15)	125 (14)	0.479
DBP, mmHg	81 (10)	82 (10)	83 (10)	84 (10)	0.426
Total cholesterol, mmol/L	5.3 (0.9)	5.3 (0.9)	5.4 (0.9)	5.4 (0.9)	0.836
HDL cholesterol, mmol/L	1.6 (0.4)	1.6 (0.4)	1.5 (0.4)	1.5 (0.3)*	0.021
LDL cholesterol, mmol/L	3.3 (0.8)	3.4 (0.9)	3.5 (0.9)	3.6 (0.9)	0.190
Triglycerides, mmol/L^a^	0.9 (0.6)	1.0 (0.5)	1.0 (0.7)	1.1 (0.6)	0.003

*Note*: The values are in mean ± standard deviation or ^a^median ± interquartile range. *p < 0.05 compared with the Low Hba1c group, **p < 0.001 compared with the Low Hba1c group, †*p* < 0.05 compared with the medium Hba1c group, || indicates standardized residuals ± 1.96.

### 3.2 Echocardiographic measures

**FIGURE 1:**
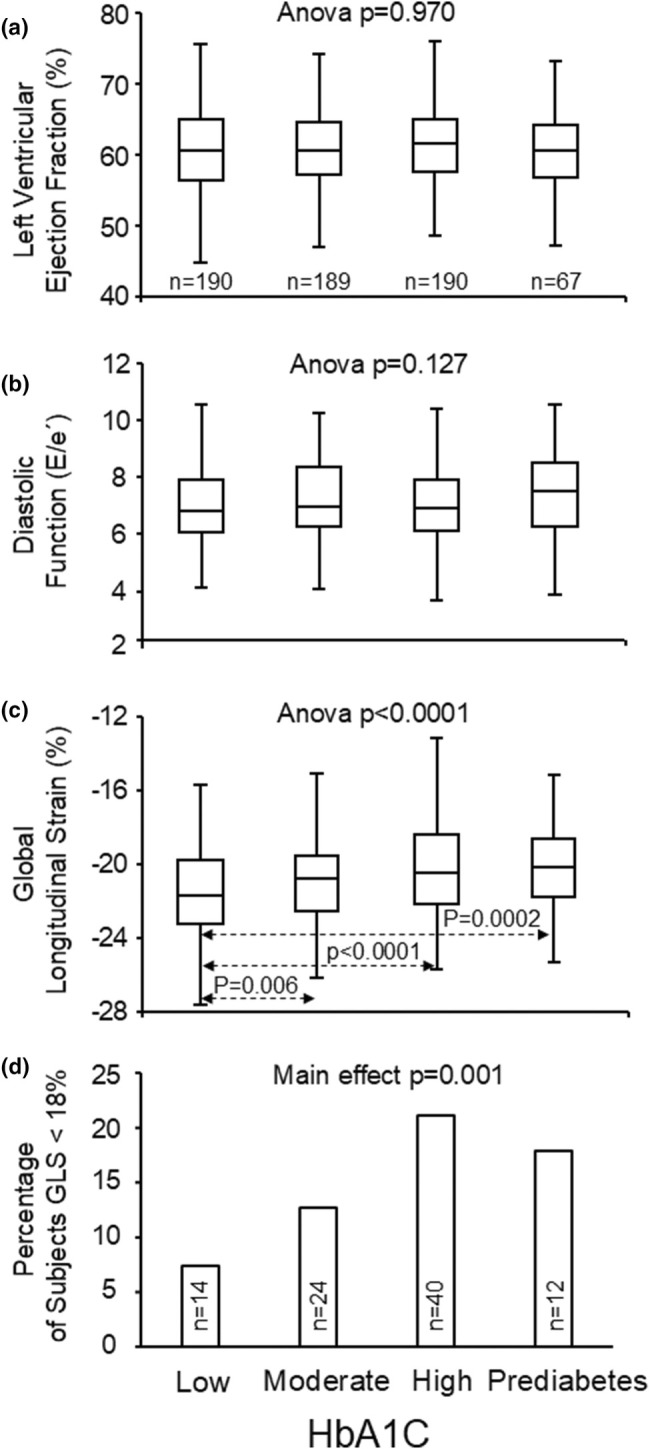
Left ventricular ejection fraction (a), diastolic function (b), global longitudinal strain (c), and the percentage and number of subjects with abnormal absolute GLS (d) according to glycated hemoglobin (HbA1c). Box and whisker plot; median (line in box), 50% of values (box), lowest and highest value (whiskers).

The relationship between HbA1c levels and echocardiographic measures is presented in Table 2. Univariate analysis showed that higher Hba1c levels were significantly associated with decreased absolute GLS and septal thickness at diastole Bonferroni's post hoc test indicated a statistically significant difference in group means of GLS between the low Hba1c tertile and the medium Hba1c tertile (*p* = 0.006), between the low tertile and the high tertile (*p* < 0.0001), and between the prediabetes group and the low Hba1c tertile (*p* = 0.0002). Cardiac structure and function in different HbA1c categories are shown in Figure 1a–c. The number of subjects with abnormal absolute GLS (<18) were 7.4%, 12.7%, 21.1%, and 17.9% (main effect *p* = 0.001) in low, medium, high HbA1c and prediabetes groups, respectively (Figure 1d). In the multivariate regression analysis, Hba1c levels were independently associated with GLS, but not with E/e' (Table 3). The most significant independent determinants of cardiac systolic function indicated by GLS were sex, BMI, Hba1c, brachial systolic blood pressure, and alcohol consumption, respectively (Table 3). The most significant independent determinants of septal thickness were sex, BMI, and systolic blood pressure, respectively (data not shown). Sex differences in cardiac remodeling are well documented; hence, gender‐specific results for males and females are provided in the Tables S1–S4.

**TABLE 2:** Echocardiographic characteristics of study population within Hba1c tertiles. One‐way ANOVA or Chi‐Square tests were used to compare the groups, followed by Bonferroni post hoc test.

	HbA1c tertiles			PreDM	*p*‐level
	Low	Medium	High		
	*n* = 190	*n* = 189	*n* = 190	*n* = 67	
Heart rate rest, bpm	65 (9)	66 (11)	68 (9)	66 (10)	0.076
LVM (ASE), g	171.9 (49.5)	179.2 (56.6)	176.4 (46.5)	181.2 (61.3)	0.485
LVM index/ASE), g/m^2^	91.0 (19.6)	94.3 (23.1)	91.8 (19.2)	93.9 (25.7)	0.432
LV end‐diastolic volume, mL	99.5 (25.8)	100.2 (25.8)	101.3 (23.0)	99.6 (23.4)	0.898
LVEDV index, mL/m^2^	52.9 (10.4)	53.2 (10.6)	53.2 (10.2)	51.8 (10.8)	0.782
Septal thickness at diastole, cm	0.90 (0.15)	0.95 (0.19)	0.94 (0.17)	0.96 (0.17)	0.026
Septal thickness index, cm/m^2^	0.49 (0.07)	0.50 (0.08)	0.50 (0.07)	0.50 (0.07)	0.085
Posterior wall thickness, cm	0.89 (0.14)	0.90 (0.16)	0.90 (0.15)	0.93 (0.14)	0.549
Posterior wall index, cm/m^2^	0.48 (0.06)	0.48 (0.07)	0.48 (0.06)	0.48 (0.06)	0.941
Relative wall thickness	0.35 (0.06)	0.35 (0.06)	0.35 (0.06)	0.36 (0.05)	0.433
LA end systolic volume, mL	55.3 (17.1)	55.8 (16.6)	55.4 (15.9)	57.9 (14.7)	0.714
LA ESV index, mL/m^2^	29.5 (7.6)	29.6 (7.5)	29.0 (7.7)	30.3 (7.1)	0.688
LVEF biplane, %	60.8 (6.0)	61.0 (5.7)	61.0 (5.9)	60.7 (5.8)	0.970
E/e'	7.1 (1.4)	7.3 (1.6)	7.1 (1.7)	7.5 (1.7)	0.127
Global longitudinal strain, %	−21.6 (2.5)	−20.8 (2.4)*	−20.4 (2.7)†	−20.1 (2.4)†	<0.001
GLS < 18%, *n* (%)	14 (7.4)||	24 (12.7)	40 (21.1)||	12 (17.9)	0.001

*Note*: *p < 0.01 compared with the Low Hba1c group, †*p* < 0.001 compared with the Low Hba1c group. || standardized residuals greater than ± 1.96.

Abbreviations: E/e´, ratio of early diastolic mitral inflow velocity to early diastolic mitral annulus velocity as a marker of diastolic function; EDV, end diastolic volume; EF, ejection fraction; ESV, end systolic volume; GLS, global longitudinal strain as a marker of systolic function; LA, left atrium; LV, left ventricle; LVM, left ventricle mass; RWT, relative wall thickness.

**TABLE 3:** The most significant determinants of cardiac function in the multivariate linear regression are based on literature (enter method) and based on stepwise method.

	GLS		E/E'	
	β	*p*‐level	β	*p*‐level
Enter method				
Adjusted *R* ^2^	0.318	<0.001	0.141	<0.001
Sex	−0.312	<0.001	0.337	<0.001
Hba1c	0.164	<0.001	0.024	0.543
BMI	0.158	<0.001	0.134	0.001
SBP	0.137	<0.001	0.236	<0.001
HDL	−0.069	0.101	−0.064	0.168
Alcohol consumption	0.073	0.043	0.093	0.022
Total cholesterol	0.050	0.172	0.030	0.457
Smoking	0.046	0.192	0.049	0.211
Stepwise method				
Adjusted *R* ^2^	0.305	<0.001	0.129	<0.001
Sex	−0.343	<0.001	0.307	<0.001
BMI	0.182	<0.001	0.159	<0.001
Hba1c	0.176	<0.001	0.034	0.372
Systolic BP	0.130	<0.001	0.229	<0.001
Alcohol consumption	0.083	0.019	0.102	0.010

*Note*: The values are presented as standardized coefficients β (per class or 1 SD) and their significance (*p*‐level).

## 4 DISCUSSION

The current study demonstrates that even within normal and prediabetic ranges, Hba1c levels are independently associated with lower absolute GLS values in the healthy middle‐aged general population. Additionally, the data suggest that Hba1c levels and prediabetes may independently contribute to the risk of subclinical left ventricular dysfunction (defined as GLS < 18%). Furthermore, our study identified sex, BMI, SBP, smoking, and alcohol consumption as the most significant predictors of LV systolic function, results that align with previous population‐based echocardiography reports (Biering‐Sørensen et al., 2017; Goncąlves et al., 2015). In addition, we tested several other potential contributors; these variables were not independently associated with GLS and were therefore excluded from the multivariable model to maintain parsimony and avoid overfitting. We acknowledge that unmeasured variables such as inflammation markers, glycemic variability, psychological stress, or genetic variation might also influence myocardial strain and warrant further investigation.

It has been established that Hba1c levels are independently associated with cardiac events and mortality (Sinning et al., 2021). Although diabetes status has been widely incorporated in the risk stratification of CVDs, continuous markers such as Hba1c have not been widely adopted for general population risk assessment. A 2021 meta‐analysis of six cohorts found a mostly monotonic association with Hba1c levels and cardiac events, like other studies that did not find a J‐shaped association (Schöttker et al., 2016; Sinning et al., 2021). Our results corroborate these findings, indicating a similar linear relationship even across normoglycemia and prediabetes for the incidence of subclinical systolic dysfunction. These observations imply a similar monotonic relationship between glycemic control and its potential underlying subclinical pathological mechanism affecting cardiac function. Several studies have previously reported an association between hyperglycemic states and lower GLS, highlighting the impact of glycemic problems on cardiac function (Huang et al., 2022; Pararajasingam et al., 2021; Skali et al., 2015; Zhou et al., 2022). In a recent study, Gao et al. demonstrated that higher HbA1c levels are independently associated with impaired GLS in T2DM patients. The authors found a near‐linear inverse relationship, even after multivariable adjustment (Gao et al., 2023). Expanding this inquiry to normoglycemia and prediabetes, our results indicate a similar relationship. However, the relationship between strain and diabetes is still somewhat controversial because some studies have not found a significant association between strain and diabetes status (Shao et al., 2020). In contrast to our results, some studies have found an association between prediabetes and LV subclinical diastolic dysfunction, instead of systolic function as in the current study. In addition to Hba1c levels and diabetes, other metabolic factors such as obesity and insulin resistance have been identified as independent factors of subclinical myocardial dysfunction (Jia et al., 2018; Ramesh et al., 2022). Relatedly, a recent study on microvascular complications in asymptomatic patients with diabetes reported that microvascular complications are associated with reduced absolute GLS, independent of atherosclerotic coronary artery disease (Pararajasingam et al., 2021), suggesting an independent relationship between cardiac function and glycemic control. Supporting this hypothesis, T2DM patients with preserved LVEF after SGLT2‐inhibitor therapy have been demonstrated to show improvement in absolute GLS (Russo et al., 2023), reinforcing the potential benefits of earlier intervention and glycemic management on cardiac function.

### 4.1 Potential mechanisms of action

While decreased elasticity of the cardiovascular system is regarded as a normal part of aging, poor glucose control could exacerbate the process via several mechanisms. Insulin resistance, a common feature in metabolic disorders, has been suggested to be an independent factor of subclinical myocardial dysfunction (Atici et al., 2021). Although we did not measure biochemical markers such as advanced glycation end‐products (AGEs), two complementary mechanisms might link higher Hba1c levels with impaired GLS. First, chronic hyperglycemia promotes the accumulation of AGEs that stiffen vasculature and the myocardial extracellular matrix, decreasing myocardial fiber shortening (Herrmann et al., 2003; McNulty et al., 2007). Second, hyperglycemia can induce a metabolic shift towards fatty‐acid utilization, increasing oxidative stress via reactive oxygen species and lipotoxicity (Ramesh et al., 2022; Sies, 2015; Tan et al., 2020). These hypotheses remain speculative in our cohort and warrant direct testing in future studies. In addition, chronic low‐grade inflammation with interstitial fibrosis, coronary microvascular dysfunction leading to hypoperfusion, and neurohormonal and epigenetic imbalance, such as increased RAAS activity and diabetes‐related miRNAs, have been suggested as factors of diabetic cardiomyopathy (Avagimyan et al., 2024; Jia et al., 2018). These processes may act synergistically with AGEs and metabolic alterations to further depress myocardial relaxation and longitudinal fiber shortening (Cao et al., 2014; Galis et al., 2024; Herrmann et al., 2003; Jia et al., 2018; McNulty et al., 2007; Ramesh et al., 2022; Salazar et al., 2021; Sies, 2015; Tan et al., 2020; Turpin et al., 2020; Tziakas et al., 2010).

### 4.2 Selective association between Hba1c and GLS

The observed selective association between Hba1c and GLS without corresponding changes in LVEF or E/e' warrants consideration of distinct physiological mechanisms behind these measures. GLS primarily reflects the longitudinal deformation of the subendocardial fibers (Algranati et al., 2011; Potter & Marwick, 2018), which are the most vulnerable to ischemia and metabolic stress, due to their higher oxygen demand and dependence on diastolic perfusion (Gul et al., 2021). These fibers contribute the most to the overall longitudinal deformation. In contrast, LVEF is a measure of global volumetric output and may remain preserved despite regional impairment due to compensation from other myocardial segments (Potter & Marwick, 2018). Similarly, E/e' reflects ventricular filling pressure, which typically increases later in the pathological progression when myocardial compliance is reduced, or interstitial fibrosis is already more advanced (Raman et al., 2009).

## 5 CLINICAL IMPLICATIONS

As the prevalence of prediabetes and cardiovascular complications is expected to increase drastically, even earlier interventions become increasingly important. Given that we found no difference in LVEF but a significant difference in GLS within normal ranges of Hba1c, GLS could be indicative of early/subclinical glycation‐induced changes in the myocardial tissue. Previously, GLS had been used in the surveillance of chemotherapy‐related cardiac dysfunction (Yang et al., 2018). However, increasing evidence suggests that GLS is a sensitive measure of cardiac function that could be relevant not only in the context of cancer therapy patients but also in diabetes care and general populations. It is evident that cardiovascular risk is multifactorial. Hba1c level could provide additional value as an independent risk factor alongside traditional risk factors such as high cholesterol levels, hypertension, and smoking. Our results indicate that high HbA1c levels could mediate adverse effects on cardiac function even within the normal reference ranges. Incorporating Hba1c into cardiovascular risk assessment, even in patients without diabetes, could be beneficial. Because the prevalence of diabetes and hyperglycemia is expected to increase drastically, this could become increasingly important. However, further research may be needed to fully understand these implications and investigate glycemic control‐related cardiac dysfunction's causality, progression, and reversibility.

## 6 LIMITATIONS

We recognize the study's cross‐sectional setting as a possible limitation regarding generalizability. Additionally, since the study is based on the Northern Finland Birth Cohort 1966, generalizing to other ethnicities, geographic locations, or age groups might be limited. While we acknowledge Hba1c as a great indicator of long‐term glycemic control, it doesn't capture short‐term fluctuations or hypoglycemic episodes, which could also impact cardiac function.

## 7 CONCLUSIONS

Within the normal and prediabetic range, Hba1c levels are independently associated with lower absolute GLS values in a healthy middle‐aged population. Hba1c levels and prediabetes may independently contribute to the risk of left ventricular subclinical dysfunction. GLS can potentially be a sensitive tool to detect early cardiac subclinical dysfunction.

## AUTHOR CONTRIBUTIONS

All authors have made substantial contributions to the conception and design of the work, and the acquisition, analysis, and interpretation of data for the work. All authors have drafted the work and reviewed it critically for important intellectual content. All authors have given final approval of the version to be published. All authors have agreed to be accountable for all aspects of the work in ensuring that questions related to the accuracy or integrity of any part of the work are appropriately investigated and resolved.

## FUNDING INFORMATION

NFBC1966 received financial support from University of Oulu Grant no. 24000692, Oulu University Hospital Grant no. 24301140, ERDF European Regional Development Fund Grant no. 539/2010 A31592, and the Finnish Foundation for Cardiovascular Research (200190, 230117).

## CONFLICT OF INTEREST STATEMENT

The results of the study are presented clearly, honestly, and without fabrication, falsification, or inappropriate data manipulation. The authors declare no conflicts of interest.

## ETHICS STATEMENT

The study was conducted according to the declaration of Helsinki and approved by the ethical committee of Northern Ostrobothnia Hospital District in Oulu, Finland. All participants provided written informed consent.

## Supporting information


Tables S1–S4.

## Data Availability

NFBC data are available from the University of Oulu, Infrastructure for Population Studies. Permission to use the data can be requested for research purposes via an electronic material request portal. In the use of data, we follow the EU General Data Protection Regulation (679/2016) and Finnish Data Protection Act. Please contact NFBC project center (nfbcprojectcenter@oulu.fi) and visit the cohort website for more information.
