# Supplementary material for: Cardiac structure and function across the continuum of glucose metabolism
Source: Physiol Rep. 2025 Jun 17;13(12):e70429. doi: 10.14814/phy2.70429 (PMC12172345; doi:10.14814/phy2.70429)
Supplement: Supplementary file 1 — Tables S1–S4. [file PHY2-13-e70429-s001.docx]

**S1.** Characteristics of males within Hba1c tertiles. One way-ANOVA or Chi-Square tests were used to compare the groups.

|  | **HbA1c tertiles** | | | |  |
| --- | --- | --- | --- | --- | --- |
|  | **Low**  **n = 85** | **Medium**  **n = 85** | **High**  **n = 85** | **PreDM**  **n = 34** | **P-level** |
| **Antropometrics** |  |  |  |  |  |
| Height, cm | 179.1 (6.0) | 178.3 (6.2) | 179.2 (5.6) | 177.4 (6.7) | 0.380 |
| Weight, kg | 83.3 (10.7) | 84.4 (13.1) | 84.5 (9.4) | 86.4 (14.0) | 0.609 |
| BMI, kg/m^2^ | 25.9 (2.8) | 26.5 (3.5) | 26.3 (2.6) | 27.5 (4.8) | 0.122 |
| Waist-to-hip ratio | 0.94 (0.05) | 0.95 (0.06) | 0.96 (0.05) | 0.98 (0.06)* | 0.004 |
| Body fat, % | 21.3 (5.2) | 22.9 (6.7) | 22.2 (5.4) | 23.1 (5.5) | 0.262 |
| Metabolic syndrome, n (%) | 18 (21.4) | 27 (33.8) | 35 (43.2) | 17 (51.5) | 0.004 |
| **Medication** |  |  |  |  |  |
| Antihypertensive agents, n (%) | 8 (9.4) | 13 (15.3) | 13 (15.3) | 5 (14.7) | 0.631 |
| Anticholesterol agents, n (%) | 1 (1.2) | 7 (8.2) | 4 (4.7) | 2 (5.9) | 0.196 |
| **Lifestyle** |  |  |  |  |  |
| Current smoker, % | 9 (10.7) | 11 (13.8) | 8 (9.9) | 5 (15.2) | 0.795 |
| Alcohol consumption, g/d | 14.9 (23.2) | 15.2 (20.2) | 16.6 (22.6) | 14.2 (17.0) | 0.942 |
| Alcohol risk use, n (%) | 8 (9.5) | 10 (12.5) | 8 (9.5) | 3 (9.1) | 0.905 |
| **Glucose metabolism** |  |  |  |  |  |
| HbA1c, mmol/mol | 31.4 (1.6) | 35.0 (1.0)** | 37.8 (0.9)** *†* | 41.2 (1.4)** *†* | <.001 |
| HbA1c % | 5.0 (0.1) | 5.4 (0.1) | 5.6 (0.1) | 5.9 (0.1) | <.001 |
| F-glucose, mmol/L | 5.2 (0.4) | 5.6 (0.5)** | 5.6 (0.4)** | 5.8 (0.49)** *†* | <.001 |
| F-insulin, mU/L | 6.8 (3.5) | 8.0 (4.2) | 9.0 (6.6)* | 9.4 (6.3) | 0.020 |
| HOMA-IR | 1.6 (1.3) | 2.1 (1.7) | 2.0 (1.7) | 2.2 (2.0) | 0.004 |
| HOMA-B | 69.3 (60.8) | 78.0 (57.3) | 76.7 (54.5) | 80 (61.3) | 0.694 |
| **Laboratory measurements** |  |  |  |  |  |
| SBP, mmHg | 127.2 (10.7) | 128.1 (12.9) | 129.3 (14.6) | 132.5 (13.8) | 0.219 |
| DBP, mmHg | 84.0 (8.6) | 84.6 (8.5) | 85.7 (9.6) | 87.3 (9.6) | 0.285 |
| Total cholesterol, mmol/L | 5.6 (1.0) | 5.4 (1.0) | 5.6 (0.9) | 5.6 (1.0) | 0.383 |
| HDL cholesterol, mmol/L | 1.4 (0.3) | 1.4 (0.3) | 1.4 (0.3) | 1.4 (0.2) | 0.325 |
| LDL cholesterol, mmol/L | 3.7 (0.9) | 3.6 (0.9) | 3.7 (0.9) | 3.8 (0.9) | 0.505 |
| Triglycerides, mmol/L | 1.17 (0.81) | 1.11 (0.63) | 1.28 (0.83) | 1.38 (0.92) | 0.325 |

**P < 0.05 compared with the Low Hba1c group, **P <0.001 compared with the Low Hba1c group, † P < 0.05 compared with the medium Hba1c group.*

**S2.** Echocardiographic characteristics of males within Hba1c tertiles. One way-ANOVA or Chi-Square tests were used to compare the groups followed by Bonferoni post-hoc test.

|  | **HbA1c tertiles** | | | |  |
| --- | --- | --- | --- | --- | --- |
|  | **Low**  **n = 85** | **Medium**  **n = 85** | **High**  **n = 85** | **PreDM**  **n = 34** | **P-level** |
| Heart rate rest, bpm | 62.7 (8.4) | 64.9 (11.8) | 65.6 (9.3) | 64.0 (9.3) | 0.240 |
| LVM (ASE), g | 208.1 (44.2) | 211.0 (58.0) | 209.9 (37.6) | 209.3 (67.0) | 0.986 |
| LVM index /ASE), g/m^2^ | 102.8 (19.2) | 103.8 (23.9) | 102.0 (18.2) | 103.9 (29.3) | 0.952 |
| LV end-diastolic volume, mL | 115.7 (25.7) | 114.4 (26.2) | 113.1 (23.7) | 109.7 (24.0) | 0.677 |
| LVEDV index, mL/m^2^ | 57.2 (11.8) | 56.6 (11.2) | 55.6 (11.0) | 54.0 (11.3) | 0.506 |
| Septal thickness at diastole, cm | 0.99 (0.13) | 1.02 (0.18) | 1.04 (0.15) | 1.04 (0.15) | 0.186 |
| Septal thickness index, cm/m^2^ | 0.49 (0.06) | 0.50 (0.07) | 0.51 (0.07) | 0.51 (0.06) | 0.217 |
| Posterior wall thickness, cm | 0.97 (0.13) | 0.97 (0.16) | 0.99 (0.12) | 0.99 (0.12) | 0.695 |
| Posterior wall thickness index, cm/m^2^ | 0.48 (0.06) | 0.48 (0.07) | 0.49 (0.06) | 0.49 (0.06) | 0.762 |
| RWT | 0.35 (0.05) | 0.36 (0.06) | 0.37 (0.06) | 0.38 (0.05) | 0.197 |
| LA end systolic volume, mL | 61.9 (19.2) | 60.5 (18.3) | 59.4 (15.1) | 60.4 (14.3) | 0.823 |
| LA ESV index, mL/m^2^ | 30.4 (8.7) | 29.7 (7.9) | 28.8 (7.1) | 30.1 (7.4) | 0.620 |
| LVEF biplane, % | 59.5 (5.7) | 60.8 (5.9) | 60.4 (6.6) | 59.7 (5.5) | 0.518 |
| E/e’ | 6.9 (1.4) | 6.9 (1.5) | 6.7 (1.2) | 7.4 (1.7) | 0.158 |
| Global longitudinal strain, % | -20.3 (2.2) | -19.8 (2.3) | -18.9 (2.5)** | -18.8 (2.4)* | <.001 |
| GLS < 18 %, n (%) | 13 (15.3) | 17 (20.0) | 33 (38.8) | 12 (35.3) | 0.001 |

Abbreviations: *LVM* left ventricle mass, *LV* left ventricle, *EDV* end diastolic volume, *RWT* relative wall thickness, *LA* left atrium, *ESV* end systolic volume, *EF* ejection fraction, *GLS* global longitudinal strain as a marker of systolic function, *E/e´* ratio of early diastolic mitral inflow velocity to early diastolic mitral annulus velocity as a marker of diastolic function. **P < 0.05 compared with the Low Hba1c group, **P <0.001 compared with the Low Hba1c group, † P < 0.05 compared with the medium Hba1c group.*

**S3.** Characteristics of females within Hba1c tertiles. One way-ANOVA or Chi-Square tests were used to compare the groups.

|  | **HbA1c tertiles** | | |  |  |
| --- | --- | --- | --- | --- | --- |
|  | **Low**  **n = 105** | **Medium**  **n = 104** | **High**  **n = 105** | **PreDM**  **n = 33** | **P-level** |
| **Antropometrics** |  |  |  |  |  |
| Height, cm | 165.4 (6.1) | 164.9 (6.1) | 165.9 (5.0) | 166.2 (5.7) | 0.573 |
| Weight, kg | 67.2 (11.2) | 69.3 (11.2) | 71.8 (13.8)* | 73.1 (12.0) | 0.017 |
| BMI, kg/m^2^ | 24.5 (3.6) | 25.5 (4.0) | 26.1 (4.6)* | 26.5 (4.3) | 0.023 |
| Waist-to-hip ratio | 0.83 (0.05) | 0.85 (0.05)* | 0.85 (0.05)** | 0.87 (0.07)** | <.001 |
| Body fat, % | 30.6 (6.7) | 31.7 (7.8) | 32.1 (7.7) | 32.7 (7.6) | 0.423 |
| Metabolic syndrome, n (%) | 2 (1.9) | 9 (8.7) | 10 (9.6) | 3 (9.1) | 0.120 |
| **Medication** |  |  |  |  |  |
| Antihypertensive agents, n (%) | 10 (9.5) | 13 (12.5) | 8 (7.6) | 5 (15.2) | 0.521 |
| Anticholesterol agents, n (%) | 0 (0) | 2 (1.9) | 2 (1.9) | 0 (0) | 0.445 |
| **Lifestyle** |  |  |  |  |  |
| Current smoker, % | 6 (5.8) | 14 (13.7) | 18 (18) | 7 (21.2) | 0.033 |
| Alcohol consumption, g/d | 5.6 (6.8) | 6.2 (8.7) | 7.0 (10.1) | 3.7 (6.2) | 0.236 |
| Alcohol risk use, n (%) | 4 (3.9) | 8 (7.8) | 10 (9.9) | 1 (3.0) | 0.280 |
| **Glucose metabolism** |  |  |  |  |  |
| HbA1c, mmol/mol | 30.5 (1.7) | 34.3 (0.9)** | 37.2 (1.1)** *†* | 40.8 (1.3)** *†* | <.001 |
| HbA1c % | 4.9 (0.2) | 5.3 (0.1) | 5.6 (0.1) | 5.9 (0.1) | <.001 |
| F-glucose, mmol/L | 4.9 (0.4) | 5.1 (0.4)* | 5.2 (0.4)** | 5.5 (0.5)** *†* | <.001 |
| F-insulin, mU/L | 5.4 (2.3) | 6.2 (3.5) | 6.8 (3.6)* | 8.0 (3.9)** *†* | <.001 |
| HOMA-IR | 1.2 (0.8) | 1.4 (1.1) | 1.4 (1.2) | 2.0 (1.5) | <.001 |
| HOMA-B | 67.8 (34.6) | 74.0 (47.6) | 68.9 (46.3) | 79.1 (53.2) | 0.070 |
| **Laboratory measurements** |  |  |  |  |  |
| SBP, mmHg | 118.1 (16.4) | 119.0 (17.7) | 119.7 (13.5) | 117.6 (11.3) | 0.847 |
| DBP, mmHg | 80.0 (10.6) | 80.4 (11.1) | 81.1 (9.6) | 80.0 (8.0) | 0.864 |
| Total cholesterol, mmol/L | 5.1 (0.7) | 5.3 (0.9) | 5.2 (0.9) | 5.3 (0.9) | 0.556 |
| HDL cholesterol, mmol/L | 1.8 (0.4) | 1.7 (0.4) | 1.7 (0.4) | 1.6 (0.3) | 0.086 |
| LDL cholesterol, mmol/L | 3.1 (0.7) | 3.3 (0.8) | 3.2 (0.8) | 3.4 (0.9) | 0.147 |
| Triglycerides, mmol/L | 0.73 (0.42) | 0.90 (0.42) | 0.88 (0.42) | 1.04 (0.46) | <.001 |

**P < 0.05 compared with the Low Hba1c group, **P <0.001 compared with the Low Hba1c group, † P < 0.05 compared with the medium Hba1c group.*

**S4.** Echocardiographic characteristics of females within Hba1c tertiles. One way-ANOVA or Chi-Square tests were used to compare the groups followed by Bonferoni post-hoc test.

|  | **HbA1c tertiles** | | | |  |
| --- | --- | --- | --- | --- | --- |
|  | **Low**  **n = 105** | **Medium**  **n = 104** | **High**  **n = 105** | **PreDM**  **n = 33** | **P-level** |
| Heart rate rest, bpm | 67.7 (8.9) | 67.1 (10.3) | 69.8 (9.0) | 67.4 (9.7) | 0.174 |
| LVM (ASE), g | 142.0 (29.7) | 152.5 (39.0) | 149.1 (33.3) | 152.2 (37.7) | 0.153 |
| LVM index /ASE), g/m^2^ | 81.2 (13.7) | 86.4 (19.1) | 83.5 (15.7) | 83.6 (16.3) | 0.170 |
| LV end-diastolic volume, mL | 86.3 (16.8) | 88.7 (18.8) | 91.9 (17.6) | 89.2 (17.7) | 0.158 |
| LVEDV index, mL/m^2^ | 49.5 (7.6) | 50.4 (9.3) | 51.3 (9.2) | 49.5 (9.9) | 0.450 |
| Septal thickness at diastole, cm | 0.83 (0.13) | 0.89 (0.16) | 0.86 (0.14) | 0.88 (0.15) | 0.066 |
| Septal thickness index, cm/m^2^ | 0.48 (0.07) | 0.50 (0.08) | 0.48 (0.07) | 0.49 (0.07) | 0.134 |
| Posterior wall thickness, cm | 0.84 (0.13) | 0.84 (0.13) | 0.84 (0.13) | 0.86 (0.12) | 0.891 |
| Posterior wall thickness index, cm/m^2^ | 0.48 (0.07) | 0.48 (0.07) | 0.47 (0.07) | 0.47 (0.05) | 0.643 |
| RWT | 0.34 (0.07) | 0.34 (0.06) | 0.34 (0.06) | 0.34 (0.05) | 0.867 |
| LA end systolic volume, mL | 49.9 (13.1) | 51.9 (14.1) | 52.2 (15.9) | 55.3 (14.9) | 0.287 |
| LA ESV index, mL/m^2^ | 28.7 (6.5) | 29.5 (7.1) | 29.2 (8.2) | 30.5 (6.8) | 0.637 |
| LVEF biplane, % | 61.9 (6.0) | 61.2 (5.5) | 61.5 (5.3) | 61.7 (6.0) | 0.820 |
| E/e’ | 7.2 (1.4) | 7.6 (1.7) | 7.5 (1.8) | 7.7 (1.8) | 0.140 |
| Global longitudinal strain, % | -22.7 (2.1) | -21.6 (2.1)* | -21.6 (2.3)* | -21.5 (1.6)* | <.001 |
| GLS < 18 %, n (%) | 1 (1.0) | 7 (6.7) | 7 (6.7) | 0 (0) | 0.065 |

Abbreviations: *LVM* left ventricle mass, *LV* left ventricle, *EDV* end diastolic volume, *RWT* relative wall thickness, *LA* left atrium, *ESV* end systolic volume, *EF* ejection fraction, *GLS* global longitudinal strain as a marker of systolic function, *E/e´* ratio of early diastolic mitral inflow velocity to early diastolic mitral annulus velocity as a marker of diastolic function. **P < 0.05 compared with the Low Hba1c group, **P <0.001 compared with the Low Hba1c group, † P < 0.05 compared with the medium Hba1c group.*
